# Supplementary material for: Establishment of soil strength in a nourished wetland using thin layer placement of dredged sediment
Source: PLoS One. 2021 May 11;16(5):e0251420. doi: 10.1371/journal.pone.0251420 (PMC8112682; doi:10.1371/journal.pone.0251420)
Supplement: Attachment — Submitted filename: PONE-D-20-25909_reviewed.pdf [file pone.0251420.s002.pdf]

ESTABLISHMENT OF SOIL STRENGTH IN A NOURISHED WETLAND USING THIN LAYER PLACEMENT OF DREDGED MATERIAL

Brian D. Harris
Graduate Research Assistant
Department of Civil and Environmental Engineering
Louisiana State University

Donnie J. Day
Graduate Research Assistant
Department of Oceanography and Coastal Science
Louisiana State University

Jack A. Cadigan
Graduate Research Assistant
Department of Civil and Environmental Engineering
Louisiana State University

Navid H. Jafari, Ph.D.
Assistant Professor
Department of Civil and Environmental Engineering
Louisiana State University

Susan E. Bailey
Research Civil Engineer
Environmental Laboratory
Engineering Research and Development Center

Zachary J. Tyler
Physical Research Scientist
Coastal Hydraulics Laboratory
Engineering Research and Development Center

A paper to be submitted for review and possible publication in *PLOS ONE*

August 18, 2020

Establishment of Soil Strength in a Nourished Wetland using Thin Layer Placement of Dredged Sediment

Brian D. Harris[1], Donnie J. Day[2], Jack A. Cadigan[1], Navid H. Jafari[1], Susan E. Bailey[3], and Zachary J. Tyler[3]

ABSTRACT: Coastal wetlands are experiencing accelerated rates of fragmentation and degradation due to ~~sea ~~sea-level rise, sediment deficits, subsidence, and salt-water intrusion. This reduces their ability to provide ecosystem benefits, such as wave attenuation, habitat for migratory birds, and a sink for carbon and nitrogen cycles. A deteriorated ~~back ~~back-barrier wetland near Avalon, New Jersey, was nourished through thin layer placement (TLP) of dredged sediment in 2016. A field investigation was conducted in 2019 using a cone penetrometer (CPT) to quantify the establishment of soil strength in a restored site compared to control sites in conjunction with traditional wetland establishment markers. Results show that the nourished area exhibited weaker strengths than the reference sites, suggesting the root system is still growing. The belowground biomass measurements correlated to the CPT strength measurements, demonstrating that shear strength measured from the cone penetrometer could serve as a surrogate to monitor wetland trajectories. During the construction process, heavily trafficked areas underwent compaction from heavy equipment loads, inhibiting the development of vegetation and highlighting how sensitive wetlands are to anthropogenic disturbances. As the need for more expansive wetland restoration projects grow, the CPT provides rapid high-resolution measurements across large sites supplying government and management agencies with vital establishment trajectories.

Keywords: thin layer placement, dredged sediment, cone penetrometer, biomass, bulk density, sleeve resistance

---

[1] Dept. of Civil and Environmental Engineering, Louisiana State University, 3355 Patrick Taylor Hall, Baton Rouge, LA 70803. bharr96@lsu.edu; jcadig1@lsu.edu; njafari@lsu.edu
[2] Dept. of Oceanography and Coastal Sciences, Louisiana State University, 3179 Energy, Coast, and Environment Building, Baton Rouge, LA 70803. dday23@lsu.edu
[3] US Army Corps of Engineers, Engineer Research and Development Center, 3909 Halls Ferry Rd, Vicksburg, MS 39180, USA. Susan.E.Bailey@usace.army.mil; Zachary.J.Tyler@usace.army.mil

INTRODUCTION

Coastal wetlands provide a variety of ~~important~~ vital ecological services, including fish and wildlife habitats, water filtration, carbon and nutrient sequestration, and flood and storm protections [1-8]. However, the combined effects of ~~sea~~ sea-level rise, hydrologic restrictions, reduced sediment supply, trenching, and subsidence coupled with extreme climatic events such as hurricanes accelerated rates of wetland fragmentation and loss, leading to the development of comprehensive coastal restoration management plans [9-18]. It is essential that restoration techniques address the elevation of the marsh platform [19], which is the overarching controlling factor on both above and belowground processes [20-22]. A potentially effective restoration technique is nourishment through thin layer placement (TLP) of dredged sediment. TLP is a method that applies dredged sediment over an existing wetland while not adversely affecting the receiving habitat's ecological functions and emulates natural accretion patterns [23-24]. TLP utilizes a pipe (usually 25 cm to 35 cm in diameter) to apply sediment across the marsh surface [25] in order to nourish marshes on the Gulf of Mexico and Atlantic coasts of the United States [13], [18], [24], [26-31]. Previous studies assessed the benefits of elevation gains through sediment placement, with a focus on vegetation response, soil conditions, and hydraulic conditions [18], [24] [26-27], [31]. However, limited information is available for the establishment of wetland soil strength concurrently with sediment distribution and consolidation post-placement, which has implications for long-term marsh resilience.

The objective of TLP is to expand wetland areas and enhance the sustainability of existing wetlands by introducing mineral and organic sediments that provide elevation gains to subsiding and eroding wetlands. The application of sediments to a deteriorating wetland increases the marsh elevation and soil aeration in the root zone~~, and~~. It increases redox potentials (E_h), resulting in the

stimulation of plant biomass and soil accretion that allow marshes to keep pace with relative ~~sea~~ sea-level rise [18], [24], [27], [32]. TLP of less than 30 cm thickness improves conditions for plant growth and marsh resiliency by increasing the soil bulk density, promoting physical stability, and vegetation establishment [23], [24], [32]. For example, in *Spartina alterniflora* dominated salt marshes, TLP increased bulk densities by 0.7 g/cm^3 [24], promoted belowground biomass establishment via rhizome development, and increased vegetation abundance and biomass in previously ponded areas [13], [26], [28], [29], [30-31]. These studies examine the ecological mechanisms associated with increased elevation gains through TLP.

Only recently has the methodology for quantifying wetland shear strength using a cone penetrometer been standardized [33], which is a ~~key~~ crucial parameter to predict resiliency against physical stressors (e.g., marsh edge erosion, collapse, uprooting) [21], [33]. As a result, the following study implements a cone penetrometer test (CPT) to measure soil strength establishment against accepted and traditional trajectory methods of measuring belowground biomass and physical index properties of a site nearly 3.5 years post nourishment. In particular, two 100 m transects were performed moving down~~-~~stream of the dredge outfall within a previously ponded section in the interior of the containment cell and along what appeared to be a higher elevation natural berm within the containment cell prior to nourishment. The results are compared to a reference site 500 m northeast of the dredge outfall. This study is the first to present the use of a CPT to define wetland restoration success and can serve as a benchmark for future restoration projects by demonstrating useful establishment monitoring practices and construction techniques for coastal stakeholders.

BACKGROUND

Overview of Avalon, NJ

The study site is located near Avalon, New Jersey, USA on the bay-side (Fig 1). The wetland is within a 17 km^2 tidal marsh complex adjacent to Great Sound. Freshwater input to the site is limited, with the nearest rivers located approximately 26 km (Great Egg Harbor River) and 56 km (Mullica River) to the north [34]. The surficial sediments are primarily comprised of Holocene era salt marsh and estuarine deposits (organic silts and clays with sand) to a maximum depth of 18 m [35-36], which pinches out from east to west [34]. The Holocene sediments are underlain by a Pleistocene sand deposit, which is approximately 37 m thick with some interbedded silts [34], [36].

Wetland degradation of the area was identified in the rapid transition of vegetated marsh platforms to un-vegetated shallow pannes through erosion and vegetation stress [17-18], [24]. The primary vegetation located across the wetland is a low-form of *S. alterniflora* that transitions to high-form *S. alterniflora* as elevation decreases near ponds and tidal creeks (Fig 1). The vegetated areas are located at an average elevation of 0.61 m NAVD88 (±0.20 m), while shallow open water pannes were found at an average elevation of 0.26 m NAVD88 (±0.13 m) [18]. The site experiences a semidiurnal tidal range of 1.39 m with an MHHW of 0.74 m NAVD88 [17]. Shallow areas were found to be continuously inundated or saturated [18]. The rapid degradation at the site prompted a restoration effort in 2016 by the United States of America Corps of Engineers (USACE) Philadelphia District (NAP).

Site Restoration

In March 2016, the USACE Philadelphia District began restoration via TLP of dredged sediment using open pipe and high-pressure discharge. In total, the restoration deposited 34,405 m^3 (45,000

yd^3) of dredged sediments from the New Jersey Intracoastal Waterway [24] across five containment sites. The sediment was contained using coir logs that were slashed after stabilization of the dredged sediment (approximately 6 months) to facilitate the containment's deterioration. Placement target elevations, based on tidal and biological references, ranged from 0.73 to 0.91 m (2.4 to 3.0 ft) NAVD88 [18], [37]. Sediment thicknesses ranged between 5.2 cm to 9.5 cm in the vegetated areas and 32.5 cm to 82.5 cm in the open water features six months after dredging was completed [18].

METHODOLOGY

Two main sections within one of the five containment cells were investigated: (1) a transect running through a previously ponded section herein referred to as "Interior", and (2) a transect running along a natural berm that separated the containment cell herein referred to as "Natural Berm" (Fig 1). Both transects extended 100 m with 20 m spacing and ~~begin~~ began at the dredge outfall and were selected to show contrasting starting points on the implications of the establishment.

Cone Penetrometer

[33] developed a portable CPT for use in ultra-soft wetland soils commonly found in coastal Louisiana that incorporates both traditional geotechnical parameters (tip resistance, sleeve friction, and pore pressure) and ecological parameters (soil moisture, electrical resistivity, and temperature) (Rewrite this sentence showing the reference somewhere in the middle). For this study, sleeve resistance is considered analogous to shear strength since the goal of this methodology is to provide comparable values as opposed to an engineering design variable. This field equipment consists of

a cone piezometer, potentiometer, and a backpack-‐mounted Data Acquisition System (DAS) capable of being manually operated by a three-person crew (Fig 2a). A modified sleeve with 5.2 cm length, perpendicular fins (Fig 2b), was used throughout this study to increase the resolution of soil resistance throughout the vegetated root mass and into the softer organic clay. Four (4) tests were conducted within 1 m^2 at each site to better capture substrata variations in root establishment.

The CPT soundings are conducted manually, with a target standard penetration rate of 2 cm/s [38]. However, the push speed can vary and occasionally stops if a stiff layer is encountered. A stop in penetration causes a drop to zero push speed (see Fig 3a) and subsequent increase in tip and sleeve resistance values (see Fig 3(b) at locations with stops) when cone penetration recommences due to soil consolidating around the CPT. A Matlab script removes these stops through the application of a Savitzky-Golay filter [39], which applies convolution to smooth the data without ~~a~~ distortion of the signal tendency [40]. The push speed in relation to unprocessed and processed sleeve resistance data is shown in Fig 3. The average push speed in Fig 3a is 1.5 cm/s. The unprocessed data (red circles in Fig. 3b) show distinct stops at 25 cm and 50 cm. The processed data (black circles in Fig 3b) remove these stops and interpolate between the less dense red circles from 10 cm to 20 cm. The paucity of data in this depth range is due to the significant force required to push through the root mat, causing the CPT to rapidly break through into the underlying soil layer at a rate faster than the sampling frequency of the data acquisition system (what does it mean?).

Field Samples

Soil samples were collected using polyvinyl chloride (PVC) cores and a Russian Peat Corer within the containment cell to validate CPT measurements with traditional wetland markers. Soil cores

were collected by inserting the PVC pipe (diameter 15 cm) to an approximate depth of 30-35 cm. Samples from these cores were field extruded and cut into 5-cm sections, bagged, shipped on ice, and stored at 4°C until processing (why 4°C, why not the field condition ambient temperature?). The samples were processed to determine belowground biomass and bulk density with depth. Four (4) Russian Peat Corer samples were taken at each site within the TLP placement area to delineate between the dredged sediment (dark gray and homogenous) and vegetated marsh sediment (brown). Fig 4 shows an example of the transition from the brown organic to dark grey mineral layer from the Interior at 60 m. The dredged sediment depth was noted and collected in bags to determine the grain size of the deposited material in accordance with [41]. Aboveground biomass was assessed by collecting triplicate 0.25 m^2 quadrats within 2 m of each CPT location. Vegetation was clipped at the soil surface, bagged, and stored on ice. Once at LSU, stem densities and heights were measured, vegetation was then dried at 60°C and weighed. The total aboveground biomass for each species was calculated as g/m^2.

RESULTS

Sediment Placement and Redistribution

This field investigation focused on one of the five restored sites. The total area of the selected site was 5.35 hectares (write square meter or kilometer). Periodic RTK-GPS elevation surveys were performed by USACE-NAP. The initial survey was performed in March 2016, prior to the restoration. Subsequent surveys were recorded immediately (within 2 weeks), 4 months, and 15 months ~~post~~ post-placement. Fig 5 shows the Kriging interpolated depth of placed dredged sediment and the magnitude of elevation loss over 15 months from the RTK-GPS elevations. The depth of sediment was determined as the difference between the pre-placement and ~~2~~ 2-week post-

placement surveys. Based on the RTK surveys, the site received between 5 and 55 cm of sediment loading. The majority of deposition was concentrated within the ponded areas with less received by the more vegetated edges of the site (Fig 5a). The amount of elevation loss was determined by the difference between the survey immediately ~~post~~ post-placement (2-~~-~~week) and the ~~15~~ 15-month survey. Region 1 contained a large pond that received more than 30 cm of sediment, which was accompanied by nearly 20 cm of elevation loss attributable to self-weight consolidation of the dredged sediment, consolidation of the wetland foundation, or displacement of soft pond sediments during the hydraulic dredging (Fig 5b). Region 2 contained a smaller pond and tidal creek, received from 5 to 25 cm of sediment, and experienced 10 to 22 cm of elevation loss. This could be attributed to losses of highly erodible and compressible sediments in this area through the tidal creek once containment was removed. Region 3 received between 10 and 20 cm of sediment but did not experience much elevation loss (1 to 11 cm). This may be due to the section not being as hydraulically connected to the nearby bay via tidal creeks like with Region 2 because of a natural berm separating the sections (Fig 1). In addition, Region 3 is near the outfall, where coarser material is likely to accumulate, whereas compressible fine-grained materials will deposit further from the outfall. The exact drivers behind elevation loss as compared to sediment thickness is not fully understood but could be an area of future study.

Directly post-nourishment, researchers from the USACE Engineering Research and Development Center (ERDC) collected dredged sediment samples in a transect from the dredge outfall through the interior (why “Interior” is capital and small in the middle of a sentence) of the containment cell to determine the gradients in grain size. Fig 6 shows the percent of fine-grained material (<0.075 mm) from their results in 2016 compared to the results from this field investigation in 2019. Immediately post nourishment, an increase in the percent of fine-grained

sediment is observed moving downstream of the dredge outfall because the coarser material was falling out of suspension faster than the finer material (i.e., silts and clays). However, the same trend is not evident in 2019, which indicates a more constant percentage of fines. This redistribution of fines is potentially attributed to vegetation-driven accretion and movement of the sediment due to hydrodynamic processes and bioturbation [31].

Interior Establishment

The Interior (Capital??) started at the dredge outfall (Fig 1) and moved northeast with measurements taken at 20 m spacing for a total of 100 m. Low-form *S. alterniflora* appeared to be the dominant vegetation type, extending the length of the outfall to 40 m. The tall-form appeared 80 m away from the outfall and was mixed with low-form at 100 m. Fig 7 shows the shear strength (τ) ± 1 standard deviation (SD) to show variability between tests with depth and dredged sediment thicknesses for each site through the Interior, and Table 1 summarizes the geotechnical and ecological data. At the time of the investigation, there was 5 cm of sediment at the dredge outfall. The shear strength increased to a peak of 159 kPa at a depth of 15 cm then decreased until the transition to the underlying soil around a depth of 29 cm. The average shear strength of the underlying soil was 43 kPa, and a slight increase was observed starting at 88 cm. At the 20 m sampling location, the total vegetation cover was approximately 95% and averaged 28 cm in height. At the time of the investigation, 5 cm of sediment was measured. The shear strength increased with depth to a peak of 172 kPa at a depth of 14 cm. The root mat transitioned to the underlying soil at a depth of 25 cm, where the average shear strength was 52 kPa. At 85 cm, an increase of shear strength was recorded and was slightly higher than at the outfall. At the 40 m sampling location, the total vegetation cover was approximately 90% and averaged 37 cm in height. At the time of the investigation, there was 10 cm of sediment at the site. The shear strength

increased with depth to a peak of 126 kPa at a depth of 21 cm. The resistance decreased until 30 cm, where it transitioned to the underlying soil with an average shear strength of 55 kPa. At a depth of 79 cm, a gradual increase in shear strength was recorded again. At 60 m, the site transitioned to a mudflat, with 37 cm of sediment. A significant strength peak was not noted and the average shear strength with depth was only 15 kPa. At 80 m, the site transitioned to a small patch of vegetation surrounded by an un-vegetated area with 29 cm of sediment. The total vegetation cover was approximately 40% and averaged 40 cm in height. The shear strength increased to 44 kPa at a depth of 13 cm and remained constant until the end of the test. At 100 m, the mudflat transitioned to a sparsely vegetated area, and the total vegetation cover was approximately 55% and averaged 62 cm in height. There was 36 cm of sediment at this site. The shear strength increased to a peak of 87 kPa at 11 cm then decreased until 22 cm to a minimum of 34 kPa. At 32 cm, a secondary spike in shear strength was recorded to a peak of 64 kPa and the influences of vegetated reinforcement was recorded down to 39 cm. As the material transitioned to the underlying soil, ~~an~~ average shear strength of 40 kPa was recorded with depth.

Table 1. Summary of vegetation and soil strength measurements for the Interior.

Site	Dredged Sediment (cm)	Above Ground Biomass (g/m^2)	Belowground Biomass			Marsh Sediment Avg. τ (kPa)
			Peak τ (kPa)	Peak Depth (cm)	Vegetation Depth (cm)	
Discharge	5	227	159	15	29	43
20 m	5	371	172	14	25	52
40 m	10	256	126	21	30	55
60 m	37	0	18	9	21	17
80 m	29	149	-	13	-	44
100 m	36	367	87	11	22	40

Natural Berm Establishment

The Natural Berm started at the dredge outfall (Fig 1) and moved east with measurements taken at

20 m spacing for a total of 100 m. Fig 8 shows the shear strength ±1 SD with depth and dredged sediment depths for each site along the Natural Berm, and Table 2 summarizes the findings. Low-form *S. alterniflora* appeared to be the dominant vegetation type, extending the length of the discharge to 100 m. The data presented at the discharge site was the same site as Interior. At the 40 m site, the total vegetation cover was approximately 95% and averaged 36 cm in height. There was 10 cm of sediment at the site. The shear strength increased to a peak of 146 kPa at a depth of 17 cm. The shear strength decreased until a depth of 47 cm as the stratigraphy transitioned to the underlying soil that had an average shear strength of 36 kPa. At the 60 m site, the vegetation began to thin out, declining to approximately 65% total cover and 27 cm in height. There was 31 cm of sediment at the site. The shear strength increased to a peak of 119 kPa at a depth of 11 cm. The end of this vegetation influence occurred at 28 cm, but a secondary shear strength spike of 79 kPa was recorded at 39 cm. The stratigraphy transitioned to the underlying soil at 59 cm to an average shear strength of 49 kPa. The shear strength with depth at this site showed more variability than at the Interior sites despite the tests being conducted on a 1 m^2 area. This secondary increase at 39 cm aligned with the depth of sediment, which could be attributed to a portion of a vegetated mat that was cut off by the flow of sediment and buried. At 80 m, the Natural Berm transitioned into a mudflat that was adjacent to a tidal creek. At the time of the investigation, there was 37 cm of sediment at the site. A slight peak in shear strength of 15 kPa was recorded at a depth of 9 cm then decreased to 9 kPa at 18 cm and remained constant with depth. At 100 m, the Natural Berm moved past the tidal creek into a sparsely vegetated site with approximately 45% total cover, averaging 28.1 cm in height, and 40 cm of sediment at the site. The shear strength increased to a peak of 84 kPa at a depth of 12 cm. The shear strength decreased to 40 kPa at a depth of 28 cm as the stratigraphy transitioned to the underlying soil.

Table 2. Summary of vegetation and soil strength measurements for the Natural Berm.

Site	Dredged Sediment (cm)	Above Ground Biomass (g/m^2)	Belowground Biomass			Marsh Sediment
			Peak τ (kPa)	Peak Depth (cm)	Vegetation Depth (cm)	Avg. τ (kPa)
Discharge	5	227	159	15	29	43
20 m	5	349				
40 m	10	254	146	17	36	36
60 m	31	215	119	11	23	49
80 m	37	0	15	9	18	11
100 m	40	162	84	12	24	40

Reference Site

Within the Reference Site, CPTs were conducted to show potential variations at low and high forms of *S. alterniflora*, with low-form being the ~~dominate~~ dominant vegetation. Due to time constraints, all coring equipment was being utilized within the nourished area so no soil cores were collected. Fig 9 shows the average shear strength values ±1 SD bands for both forms of vegetation. For the low-form, the peak shear strength of 263 kPa was found at a depth of 11 cm, and the vegetation influence was recorded down to 40 cm. The underlying soil showed an average shear strength of 46 kPa. The high-form of *S. alterniflora* was found at a lower elevation than the low-form~~,~~ within a shallow confined pond. The peak shear strength of 123 kPa was found at a depth of 11 cm, and the vegetation influence was recorded down to 30 cm. The underlying soil exhibited an average shear strength of 46 kPa. The tall-form root strength was weaker than the low-form likely due to the lower elevation resulting in a longer inundation period. The effect of roots on strength was only observed down to 30 cm for the tall-form when compared to the low-form of 40 cm root zone. Within the nourished site, the shear strength values were higher than the high-form of *S. alterniflora* at the reference site but were significantly less than the peak shear strength experienced within the low-form *S. alterniflora*. In addition, the peak shear strength was found at a deeper depth than the two control plots, which could signify that the vegetation roots have yet to

fully establish themselves within the dredged sediment. Overall, the average underlying soil shear strength was 39 kPa for the nourished site. If the two mudflat sites were removed, it increases to 45 kPa, consistent with the reference site. This shows that the underlying soil exhibits an intrinsic strength that is consistent throughout the nourished and control areas and not affected by surface wetland functions.

Biophysical Drivers

To validate the effectiveness of the CPT against biophysical drivers, the belowground biomass, bulk density, and moisture content were measured at each vegetated site and compared to the shear strength profile. Fig 10 shows that shear strength and belowground biomass exhibited analogous trends with depth, increasing from the marsh surface to a depth of 13 cm where peak belowground biomass is located and then a similar decrease trend with depth to 30 cm.

Fig 11 shows the summary of shear strength as it relates to belowground biomass measurements. Lower amounts of belowground biomass were found within the dredged sediment, which corresponds to lower shear strengths because the CPT ~~is measuring~~measures the strength of the TLP sediment. It is postulated that as the dredged sediment is further established, the presence of belowground biomass will increase the strength to resemble the measurements within the native “marsh sediment”.

Fig 12 show a summary of shear strength as it relates to belowground biomass, bulk density, and moisture content plots, divided into varying thicknesses of dredged sediment: ≤5 cm, 5–10 cm, 10–30 cm, and >30 cm. The greatest shear strength values of 160 kPa were found in areas of ≤5 cm dredged sediment thickness with a peak depth of 13 cm, and the lowest shear strength values at peak depth were in areas >30 cm at across both the Interior and Natural Berm sites (Fig 12a).

The shaded regions in the CPT profiles illustrate the variability, and it signifies that the <5 cm and 5-10 cm thicknesses exhibit less scatter (i.e., uncertainty). This may be attributed to vegetated wetlands with similar biomass productivity. As the thickness of TLP increases, the shaded regions increase. This signifies the shear strength in the dredged sediment is significantly more variable because of a wide range of particle gradation (sand to clay) and different levels of biomass productivity. Another key observation of Fig 12a is that the peak strength and belowground biomass decrease with increasing TLP thickness, which suggests live roots and rhizomes may be providing ~~the~~ a sharp increase in strength. The greatest belowground biomass contribution of ~0.8 g/cm^3 was found in areas of that received ≤5 cm of dredged sediment (Fig 12b). The same trend was observed in the 5 to 10 cm thickness but with lower belowground biomass values. In contrast, the lowest belowground biomass contribution of 0.01 g/cm^3 was found in areas of that received greater than 30 cm TLP because of their lower elevation and hence more deteriorated vegetation. The transition between the differing belowground biomass profiles occurs at or near 10 cm of TLP. This means that there is a tipping point that the geotechnical properties will resemble vegetated wetlands or inorganic sediments from the TLP. This information can guide the future trajectory of the specific wetland if the TLP thickness is measured. Bulk densities were highest in areas that received >30 cm TLP, which is consistent to bulk densities of inorganic sediment [42]. The bulk densities for less than 10 cm TLP exhibit ~~organic~~ organic-rich soils [43]. At higher TLP thicknesses, bulk densities are approximately 0.6 g/cm^3 near the surface for about the first 15 cm, but gradually decrease to bulk densities similar to that of the < 10 cm TLP (Fig 12c). This behavior could be a result of mixing of the dredged sediment with native sediment and provides another means to understand the trajectory of TLP. The moisture content [*w (%)*] behaves in direct contrast to bulk density because organic material inherently contains more pore water. The moisture

content tends to increase with depth to more than 500% for the ≤5 cm and 5–10 cm sites, yet remained at constant with depth in the areas that received >30 cm of sediment (Fig 12d). These higher moisture contents are a key indicator of greater biomass establishment in areas receiving less than 10 cm of sediment whereas greater sediment inputs have lower moisture contents due to lower concentrations of biomass.

Compaction

During the investigation, tracks of ponded marsh were found throughout the area (Fig 1). CPTs were conducted within the ponded tracks (4) and the directly adjacent vegetated zones (6). Fig 13 shows the tip resistances (q_t) with depth for both conditions alongside a picture of the test area after a CPT was completed. In the vegetated zones, the q_t increases to a peak resistance before decreasing at the underlying soil. However, the ponded zones indicate that the peak q_t occurs at the soil surface and then decreases with depth, exemplifying the site was disturbed through compaction. During the nourishment of the site, this was a common path for marsh buggies carrying the coir logs and dredging pipe, and the effects of the continued compaction are still prevalent within the marsh 3.5 years later. [44] Documented how compaction induced via heavy machinery during the restoration of a wetland results in negative impacts on root development and biomass production, which was attributed to the increase in bulk density inhibiting root penetration [45]. Fig 13 demonstrates how sensitive wetland environments are to anthropogenic disturbances. Careful consideration is needed during planning and construction to minimize lasting negative impacts.

DISCUSSION

Prior to the TLP nourishment, this site was highly degraded as vegetated portions of wetlands rapidly transitioned to un-vegetated shallow pannes. This downward shift of elevation relative to sea-~~-~~level induces higher levels of stress on the vegetation health, lowering the wetland resistance to physical stressors. This is evident in the lower shear strength of the high-form of *S. alterniflora* compared to the low-form, which resides at a higher elevation in the tidal regime (i.e. lower inundation periods). The rate of ~~sea~~ sea-level rise in this area is 1 cm/yr [46], while accretion is 0.3 cm/yr [47], leaving an elevation deficit of -0.7 cm/yr. The average elevation gain from this TLP nourishment increased the marsh elevation by 24 cm, indicating that this elevation deficit is offset by 34 years.

Fig 14 shows a conceptual model of the trajectory of wetland strength and relative elevation pre- and post-sediment nourishment. Stage 1 represents a deteriorated phase with mean low water levels at or near the marsh surface, and low shear strengths that ~~was~~ were recently nourished with sediment. This nourishment occurs over the time span of weeks to months depending on the size of the area and dredger capabilities. As time progresses, the site will transition to Stage 2, which is characterized by the initial establishment of the native vegetation bringing with it increases in shear strength. However, over this ~~time span~~period of months to years, the marsh surface elevation will decrease as the dredged sediment dewaters and experiences self-weight consolidation and as the foundation consolidates under the load of the TLP [48]. Stage 3 represents a fully established wetland and is characterized by peak above and belowground vegetation and shear strengths. This transition occurs over the course of years, and the marsh surface will experience elevation gains due to sediment accretion [27], [43], [49-51]. Because SLR is greater than accretion rates, the site will eventually transform to a deteriorated wetland (Stage 4), similar to this field study site prior to nourishment [52]. This progression spurred by SLR outpacing sediment accretion and increasing

inundation periods will occur over the course of decades. The increase in inundation period will negatively impact the wetland health and overall strength. The primary objective of sediment nourishment is to provide the essential increase in marsh surface elevation, allowing for a more productive wetland that can use accretion and sediment capture to help bolster elevation. However, SLRs will eventually overtake the wetland surface. TLP is not a permanent solution to wetland management but serves as critical periodic maintenance. The conceptual diagram in Fig 14 provides a framework to quantify time-scales of trajectories (establishment and deterioration) and the corresponding shear strengths. This can be applied by coastal managers to better understand the efficacy of TLP and prioritize sites for restoration.

CONCLUSIONS

This field investigation was performed using co-located cores and cone penetrometer tests to measure wetland strength establishment ~~post~~ post-TLP nourishment. These measurements were compared to biophysical drivers across two transects within a previously interior pond and along a natural berm. The primary findings of this study are summarized as follows:

- The nourished site exhibited weaker shear strength within the vegetated mat when compared to the reference area, suggesting that the nourished site is still establishing vegetation root structure.
- Belowground biomass and CPT shear strength measurements correlated with depth, demonstrating that this methodology can provide accurate quantifications on site development trajectory in a more streamlined and less intrusive manner than traditional ecological and geotechnical techniques.
- The underlying soil below the roots exhibits an intrinsic strength that is consistent

throughout both restored and reference areas and are not affected by surface wetland functions and is likely a function of the long-term geological history of the site.

- Sediment sampling directly post-nourishment and 3.5 years later showed a redistribution of grain size gradients, which can be attributed to coastal processes reworking the sediments, accretion, or bioturbation.
- Heavily used construction tracks compacted a small area of the marsh platform, inhibiting the establishment of vegetation 3.5 years ~~post~~ post-construction.
- A conceptual model outlining the stages of TLP, wetland strength establishment, full site establishment, and the subsequent deterioration is presented.

The results presented herein capture one point in the establishment process of a sediment-nourished wetland, and future monitoring is necessary to fully understand the long-term impacts of TLP on marsh resilience and maintenance. While other meaningful studies utilize extended growing seasons to understand the long-term effects of sediment placement on the biological, chemical, and physical dependencies, this field investigation demonstrated the usefulness of the cone penetrometer when evaluating wetland strength establishment. The utilization of the cone penetrometer in combination with traditional wetland markers, can provide rapid assessments of wetland status for coastal restoration and management practices. For this specific case, baseline data would have provided additional information to better indicate wetland establishment trajectories and thus is highly recommended for future field investigations.

ACKNOWLEDGEMENTS (Conflicting with the Financial Disclosure Statement)

Support for this research was provided in part by the Engineering Research and Development

Center (ERDC) through the USACE Dredged Operations and Environmental Research (DOER) program, the Department of Defense (DoD) through its Science, Mathematics, and Research for Transformation (SMART), and the Resources and Ecosystems Sustainability, Tourist Opportunities, and Revived Economies of the Gulf Coast States Act (RESTORE Act) center of excellence grant. In addition, the authors would like to thank Jacob Berkowitz and Christine VanZomeren from ERDC's Environmental Lab and the following LSU students for assisting with field and laboratory testing: Dominion Ajayi, Mahajebin Haque, Cameron Markowitz, and Amina Meselhe.

REFERENCES

1. Mitsch WJ, Gosselink JG. Wetlands (Second Edition). New York: Wiley. 1993.
2. Costanza R, d'Arge, R, de Groot R, Farber S, Grasso M, Hannon B, Limburg K, Naeem S, O'Neill RV, Paruelo J, Raskin RG, Sutton P, van den Belt M. The value of ecosystem services: Pputting the issues in perspective. Ecol. Econ. 1998. 25 (1), 67–72.
3. Mitsch WJ, Gosselink JG. The value of wetlands: Iimportance of scale and landscape setting. Ecological Economics. 2000. 35(1): 25-33.
4. Day JW, Christian RR, Boesch DM, Yáñez-Arancibia A, Morris J, Twilley RR, Naylor L, Schaffner L. Consequences of climate change on the ecogeomorphology of coastal wetlands. Estuaries and Coasts. 2008. 31(3): 477-491.
5. Reddy, KR, DeLaune CBC. Nutrients in Wetlands: Implications to Water Quality under Changing Climatic Conditions. 2010. (what is the relation of this article with the current study?
6. Barbier EB, Hacker SD, Kennedy C, Koch EW, Stier AC, Silliman BR. The value of estuarine and coastal ecosystem services. Ecological Monographs. 2011. 81(2): 169-193.
7. Twilley RR, Bentley SJ Sr., Chen Q, Edmonds DA, Hagen SC, Lam NSN, Willson CS, Xu K, Braud D, Hampton PR, McCall A. Co-evolution of wetland landscapes, flooding, and human settlement in the Mississippi River Delta Plain. Sustainability science. 2016. 11(4): 711-731.
8. CPRA (Coastal Protection and Restoration Authority). Louisiana's comprehensive master plan for a sustainable coast. Baton Rouge, LA: CPRA. 2017.

9. Davis GH. Land subsidence and sea level rise on the Atlantic Coastal Plain of the United States. Environmental Geology and Water Science. 1997. 10, 67–80.
10. Turner RE. Wetland loss in the Northern Gulf of Mexico: Multiple working hypotheses. 1997. 20(1): 1-13.
11. Day J, Britsch L, Hawes S, Shaffer G, Reed D, Cahoon D. Pattern and Process of Land Loss in the Mississippi Delta: A Spatial and Temporal Analysis of Wetland Habitat Change. Estuaries 2000. 23: 425-438.
12. Costa-Pierce B, Weinstein M. Use of dredge materials for coastal restoration. Ecological Engineering. 2002. 19.
13. Stagg C, Mendelssohn I. Controls on resilience and stability in a sediment-subsidized salt marsh. Ecological applications: a publication of the Ecological Society of America. 2011. 21: 1731-1744.
14. Kulp M, Penland S, Williams SJ, Jenkins C, Flocks J, Kindinger J. Geologic Framework, Evolution, and Sediment Resources for Restoration of the Louisiana Coastal Zone. Journal of Coastal Research. 2005. 56-71. 29.
15. Saleh F, Weinstein MP. The role of nature-based infrastructure (NBI) in coastal resiliency planning: A literature review. Journal of Environmental Management. 2016. 183: 1088-1098.
16. Berkowitz JF, Vanzomeren CM, Piercy CD. Marsh restoration using thin layer sediment addition: Initial soil evaluation. Wetland Science and Practice. 2017. 34.
17. Berkowitz JF, Vanzomeren CM, Piercy CD, White JR. Evaluation of coastal wetland soil properties in a degrading marsh. Estuarine, Coastal and Shelf Science. 2018. 212: 311-317.

18. VanZomeren CM, Berkowitz JF, Piercy CD, White JR. Restoring a degraded marsh using thin layer sediment placement: Short term effects on soil physical and biogeochemical properties. Ecological Engineering. 2018. 120: 61-67.

19. Broome SW, Seneca ED, Woodhouse WW. Tidal salt marsh restoration. Aquatic Botany. 1988. 32(1): 1-22.

20. Morris JT, Sundareshwar PV, Nietch CT, Kjerfve B, Cahoon DR. Responses of Coastal Wetlands to Rising Sea Level. Ecology. 2002. 83(10): 2869-2877.

21. Day JW, Kemp GP, Reed DJ, Cahoon DR, Boumans RM, Suhayda JM, Gambrell R. Vegetation death and rapid loss of surface elevation in two contrasting Mississippi delta salt marshes: The role of sedimentation, auto-compaction and sea-level rise. Ecological Engineering. 2011. 37(2): 229-240.

22. Morris JT, Sundberg K, Hopkinson CS. Salt Marsh Primary Production and Its Responses to Relative Sea Level and Nutrients in Estuaries at Plum Island, Massachusetts, and North Inlet, South Carolina, USA. Oceanography. 2013. 26(3): 78-84.

23. Wilber P. Thin-layer disposal: Concepts and terminology. Environmental Effects of Dredging Information Exchange Bulletin D-92-1. Vicksburg, MS: U.S. Army Engineer Waterways Experiment Station. 1992.

24. Berkowitz JF, Vanzomeren CM, Piercy CD, Keys TA. Marsh Restoration Using Thin Layer Placement: Soil and Hydrologic Response to Direct Sediment Application. 2019.

25. Ray G. Thin Layer Placement of Dredged Material on Coastal Wetlands: A Review of the Technical and Scientific Literature. ERDC/EL TN-07-1. US Army Corps of Engineers. Vicksburg MS. 2007.

26. Reimold RJ, Hardisky MA, Adams PC. The effects of smothering a Spartina alterniflora salt marsh with dredged material. Dredged Material Research Program Technical Report D-78-38. U.S. Army Engineer Waterways Experiment Station, Vicksburg, MS. 1978.

27. DeLaune RD, Pezeshki SR, Pardue JH, Whitcomb JH, Patrick WH. Some influences of sediment addition to a deteriorating salt marsh in the Mississippi River Deltaic Plain: A pilot study. Journal of Coastal Research. 1990. 6:181-188.

28. Ford MA, Cahoon DR, Lynch JC. Restoring marsh elevation in a rapidly subsiding salt marsh by thin-layer deposition of dredged material. Ecological Engineering. 1999. 12(3-4): 189-205.

29. Mendelssohn I, Kuhn NL. Sediment subsidy: effects on soil-plant responses in a rapidly submerging coastal salt marsh. Ecological Engineering. 2003. 21:115-128.

30. Slocum MG, Mendelssohn IA, Kuhn NL. Effects of Sediment Slurry Enrichment on Salt Marsh Rehabilitation: Plant and Soil Responses over Seven Years. Estuaries. 2005. 28(4): 519-528.

31. Croft AL, Leonard LA, Alphin T, Cahoon LB, Posey M. The effects of thin layer sand renourishment on tidal marsh processes: Masonboro Island, North Carolina. Estuaries and Coasts. 2006. 29:737-750.

32. Baustian J, Mendelssohn I. Hurricane-Induced Sedimentation Improves Marsh Resilience and Vegetation Vigor under High Rates of Relative Sea Level Rise. Wetlands. 2015. 35: 1-8.

33. Jafari NH, Harris BD, Cadigan JA, Chen Q. Piezocone penetrometer measurements in coastal Louisiana wetlands. Ecological Engineering. 2019. 127: 338-347.

34. Ferland MA. Holocene depositional history of the Southern New Jersey barrier and backbarrier regions. USACE, ed., USAEWES Coastal Engineering Research Center, Vicksburg, MS. 1990.

35. Newell WL, Powars DS, Owens JP, Schindler JS. Surficial geologic map of New Jersey; southern sheet. Open-File Report. 1995.

36. Sugarman PJ, Monteverde DH, Standford SD, Johnson SW, Stroiteleva Y, Pristas RS, Vandegrift K, Domber SE. Geologic and Aquifer Map of Cape May County, New Jersey. Department of Environ. Protection Water Resources Management New Jersey Geological and Water Survey. 2016.

37. Piercy CD, Welp T, Chasen MA. Avalon New Jersey dredged material thin layer placement project. Scientific Group of the London Convention, 40th Meeting. 27- 31 March 2017.

38. Robertson PK, Cabal KL. Guide to Cone Penetration Testing for Geotechnical Engineering. Gregg Drilling & Testing, Inc. 2015. 6th.

39. Savitzky A, Golay MJE. Smoothing and Differentiation of Data by Simplified Least Squares Procedures. Analytical Chemistry, 1964. 36(8), 1627-1639.

40. Cadigan JA, Harris BD, Jafari NH. jcadig1/LSU_CPT_Processing: LSU_CPT_Processing-v1.0 (Version v1.0). Zenodo. 2020, March 31. http://doi.org/10.5281/zenodo.3734396

41. ASTM D6913 / D6913M-17, Standard Test Methods for Particle-Size Distribution (Gradation) of Soils Using Sieve Analysis, ASTM International, West Conshohocken, PA. 2017. www.astm.org

42. Holtz RD, Kovacs WD, Sheahan TC. An Introduction to Geotechnical Engineering. Prentice Hall, Upper Saddle River. 2011.

43. Morris JT, Barber DC, Callaway JC, Chambers R, Hagen SC, Hopkinson CS, Johnson BJ, Megonigal P, Neubauer SC, Troxler T, Wigand C. Contributions of organic and inorganic matter to sediment volume and accretion in tidal wetlands at steady state. Earth's Future. 2016. 4(4): 110-121.

44. Fenstermacher DE, Rabenhorst MC, Lang MW, McCarty GW, Needelman BA. Carbon in Natural, Cultivated, and Restored Depressional Wetlands in the Mid-Atlantic Coastal Plain. Journal of Environmental Quality. 2016. 45(2): 743-750.

45. Shierlaw J, Alston AM. Effect of soil compaction on root growth and uptake of phosphorus. Plant and Soil. 1984. 77(1): 15-28.

46. Miller KG, Kopp RE, Horton BP, Browning JV, Kemp AC. A geological perspective on sea-level rise and its impacts along the U.S. mid-Atlantic coast, Earth’s Future. 2013. doi:10.1002/2013EF000135.

47. Velinsky D, Sommerfield C, Enache M, Charles D. Nutrient and Ecological Histories in Barnegat Bay, New Jersey. 2011. PCER Report No. 10-15.

48. Graham SA, Mendelssohn IA. Functional assessment of differential sediment slurry applications in a deteriorating brackish marsh. Ecological Engineering. 2013. 51: 264-74.

49. Turner RE, Swenson EM, Milan CS. Organic and inorganic contributions to vertical accretion in salt marsh sediments. 2001. Pp. 583-595 In.: M. Weinstein and K. Kreeger (eds.). Concepts and Controversies in Tidal Marsh Ecology. Kluwer Academic Publishing, Dordrecht, Netherlands. 864 pp.

50. Neubauer SC. Contributions of mineral and organic components to tidal freshwater marsh accretion. Estuarine, Coastal and Shelf Science. 2008. 78(1): 78-88.

51. Quirk T, Graham SA, Mendelssohn IA, Snedden G, Day JW, Twilley RR, Shaffer G, Sharp LA, Pahl J, Lane RR. Mississippi river sediment diversions and coastal wetland sustainability: Synthesis of responses to freshwater, sediment, and nutrient inputs. Estuarine, Coastal and Shelf Science. 2019. 221: 170-183.

52. Nyman JA, DeLaune RD, Roberts HH, Patrick WH. Relationship between vegetation and soil formation in a rapidly submerging coastal marsh. Marine Ecology Progress Series. 1993. 96(3): 269-279.
