# Supplementary material for: Establishment of soil strength in a nourished wetland using thin layer placement of dredged sediment
Source: PLoS One. 2021 May 11;16(5):e0251420. doi: 10.1371/journal.pone.0251420 (PMC8112682; doi:10.1371/journal.pone.0251420)
Supplement: Attachment — Submitted filename: Reviewer Comments_10092020.docx [file pone.0251420.s003.docx]

Dear Associate Editor,

This manuscript addresses soil strength and biophysical factors of thin layer placement as a restoration technique for degraded marshes. The manuscript describes the use of a cone penetrometer as a proxy for soil strength along two transects, a heavy machinery impacted zone, and a control area. Overall, the manuscript addresses important data gaps in methodology and marsh restoration trajectories following thin layer placement. However, the manuscript requires additional clarification and text in several locations, and mainly in the discussion section. I am recommending major revisions to this manuscript, which upon completion will provide important new data to the wetland literature. Specific comments are provided below.

60	Clarify “traditional wetland establishment markers” to specify belowground biomass, bulk density, and moisture content. Are these parameters only related to wetland establishment, or more broadly applicable to wetland condition?

61	This statement is a little misleading. Based on the site description, large areas were un-vegetated prior to thin layer placement. Post thin layer placement, these areas are beginning to vegetate. Is a comparison between a vegetated control area and an un-vegetated open treatment area valid?

80	The use of “nourishment” is also misleading. Are you nourishing elevation? The objective of the thin layer placement is to increase elevation. A secondary effect is nutrient addition (i.e. “nourishment”) from the dredged material.

85	There are examples of thin layer placement on the West coast, and should be included in this section. Specifically, Seal Beach, CA is an example of a West Coast TLP project.

92	Is there really a significant contribution of organic material from the dredged sediment?

102	Add a transition here; the change in ecological discussion to shear strength is abrupt.

129	The literature uses “short form” and “tall form” of S. alterniflora.

154	Establishment of what?

157	Is ultra-soft wetland soils analogous to unconsolidated soils? If so, include unconsolidated soils as an additional term that people are familiar with. If not, define what “ultra-soft” refers to.

204	Consolidation of the dredged material is expected. However this is not addressed until much father in the text. Recommend this information is moved up in the text to clarify that elevation loss was not un-expected.

231	Vegetation driven accretion? The accumulation of organic material through vegetation growth redistributes fine materials? More likely redistribution of fine material is through hydrodynamic processes and bioturbation; above ground biomass would increase fine material deposition. Clarification of this statement is needed.

265	Table 1. What does Vegetation Depth (cm) describe? The original marsh surface? Clarification is needed.

326	Clarify what “dredge sediment is further established” means. Vegetation establishment within the placement sediment?

361	Correct biomass to organic material.

365	Tracks of what?

308	The discussion section is very thin. One important point to discuss is open water areas that received dredged material in the context of the vegetated control areas. These are not directly comparable, yet offer an opportunity to discuss the trajectory of an open water area that receives dredge material moving towards shear strength of a vegetated area. Additionally, a discussion of Avalon marsh trajectory is missing, and perhaps is not possible given one sampling date? How does this fit into the conceptual model? Going back to the objectives outlined in 1121-115, was this wetland restoration project a “success”? How do you define success with one sampling date with CPT? There seems to be two competing objectives of this paper: 1. how does the TLP site compare to the reference area and 2. use of the CPT as a methodology to measure restoration success and trajectory. Revise the discussion section, and throughout as needed, to address these two objectives.

387	Does this calculation consider additional potential elevation gains from organic matter accumulation and resulting accretion from healthy vegetation growth?
