# Supplementary material for: Establishment of soil strength in a nourished wetland using thin layer placement of dredged sediment
Source: PLoS One. 2021 May 11;16(5):e0251420. doi: 10.1371/journal.pone.0251420 (PMC8112682; doi:10.1371/journal.pone.0251420)
Supplement: Attachment — Submitted filename: PONE-D-20-25909_Response_to_Reviewers.docx [file pone.0251420.s004.docx]

**Response to Reviewer Comments**

**PLOS ONE**

**Paper ID:**	D-20-25909

**Title:**	Establishment of Soil Strength in a Nourished Wetland Using Thin Layer Placement of Dredged Material

**Authors:**	Brian Harris, Ph.D., Donnie Day, Jack Cadigan, Navid H. Jafari, Ph.D., Susan Bailey, and Zachary Tyler

**Date:**	January 23, 2021

| **Editor-in-Chief Comments** | **Authors’ Responses** |
| --- | --- |
| Thank you for submitting your manuscript to PLOS ONE. After careful consideration, we feel that it has merit but does not fully meet PLOS ONE’s publication criteria as it currently stands. Therefore, we invite you to submit a revised version of the manuscript that addresses the points raised during the review process.  In the process of making your revisions, please pay careful attention to the comments from Reviewers 2 and 3 about identifying the goals/objectives of the project, broadening the scope by referencing additional literature from a broader geographic range, and using standard (or less-regionally specific) terminology that is common to the field of wetland restoration. By addressing their comments, there is an opportunity to reframe, and more clearly link, the introduction and discussion sections and to clarify restoration goals and metrics for success. | The authors appreciate the Editor-in-Chief’s invitation to revise the manuscript. Thank you. |

| **Journal Requirements** | **Authors’ Responses** |
| --- | --- |
| Thank you for including the following ethics statement on the submission details page:  'Field work was conducted on land overseen by the Wetlands Institute (Avalon, NJ). Contact: Lenore Tedesco'  * In the Methods section of your manuscript, please amend the ethics statement to confirm that the name institution approved the field site access and sampling collection. | The Methodology section was revised to include this:  “This field access and sampling collection was approved and overseen by the Wetlands Institute.” |
| Thank you for stating the following in the Acknowledgments Section of your manuscript:  Support for this research was provided in part by the Engineering Research and Development Center (ERDC) through the USACE Dredged Operations and Environmental Research (DOER) program, the Department of Defense (DoD) through its Science, Mathematics, and Research for Transformation (SMART), and the Resources and Ecosystems Sustainability, Tourist Opportunities, and Revived Economies of the Gulf Coast States Act (RESTORE Act) center of excellence grant. In addition, the authors would like to thank Jacob Berkowitz and Christine VanZomeren from ERDC’s Environmental Lab and the following LSU students for assisting with field and laboratory testing: Dominion Ajayi, 457 Mahajebin Haque, Cameron Markowitz, and Amina Meselhe."  i) We note that you have provided funding information that is not currently declared in your Funding Statement. However, funding information should not appear in the Acknowledgments section or other areas of your manuscript. We will only publish funding information present in the Funding Statement section of the online submission form.  ii) Please remove any funding-related text from the manuscript and let us know how you would like to update your Funding Statement. Currently, your Funding Statement reads as follows:  "The author(s) received no specific funding for this work".   * Please include your amended statements within your cover letter; we will change the online submission form on your behalf | The reference to funding within the acknowledgements section has been removed because no direct funding was provided:  “The authors would like to thank support offered by the Engineering Research and Development Center (ERDC) through the USACE Dredged Operations and Environmental Research (DOER) program…” |
| We note that Figures [1 and 5] in your submission contain [map/satellite] images which may be copyrighted. All PLOS content is published under the Creative Commons Attribution License (CC BY 4.0), which means that the manuscript, images, and Supporting Information files will be freely available online, and any third party is permitted to access, download, copy, distribute, and use these materials in any way, even commercially, with proper attribution. For these reasons, we cannot publish previously copyrighted maps or satellite images created using proprietary data, such as Google software (Google Maps, Street View, and Earth). For more information, see our copyright guidelines: <http://journals.plos.org/plosone/s/licenses-and-copyright>.   We require you to either (1) present written permission from the copyright holder to publish these figures specifically under the CC BY 4.0 license, or (2) remove the figures from your submission: | Thank you for bringing this to our attention. The imagery used within Fig. 1 has been cited within the image. The direct links to Fig. 1 have been provided below. The image used was the USGS The National Map: Orthoimagery. Data refreshed October 2020.  <https://apps.nationalmap.gov/viewer/>  The previous Figure 5 has been removed from the manuscript. |
| Please amend either the title on the online submission form (via Edit Submission) or the title in the manuscript so that they are identical. | The title on the manuscript has been corrected. |
| We note that Figure [2] includes an image of a participant in the study.  As per the PLOS ONE policy ([http://journals.plos.org/plosone/s/submission-guidelines#loc-human-subjects-research](about:blank#loc-human-subjects-research)) on papers that include identifying, or potentially identifying, information, the individual(s) or parent(s)/guardian(s) must be informed of the terms of the PLOS open-access (CC-BY) license and provide specific permission for publication of these details under the terms of this license. Please download the [Consent Form for Publication in a PLOS Journal](about:blank) ([http://journals.plos.org/plosone/s/file?id=8ce6/plos-consent-form-english.pdf](about:blank)). The signed consent form should not be submitted with the manuscript, but should be securely filed in the individual's case notes. Please amend the methods section and ethics statement of the manuscript to explicitly state that the patient/participant has provided consent for publication: “The individual in this manuscript has given written informed consent (as outlined in PLOS consent form) to publish these case details”. | There are 3 people in this picture: Zachary Tyler (co-author), Jacob Berkowitz, and Brian Harris (author). All have signed consent forms. In addition, the required statement as been added to the manuscript. Thank you. |

| **Additional Editor Comments** | **Authors’ Responses** |
| --- | --- |
| This manuscript on the establishment of soil strength using thin layer placement requires major revisions to address overarching concerns and specific comments identified during the review process. Please carefully consider the comments and recommendations for revision that the reviewers have made, especially as they pertain to the scope and terminology used to describe the project and its implications for wetland restoration. For instance, utilizing standard terminology for nourishment and thin layer placement projects and expanding the literature cited to encompass broader geographic range will help reach the broader audience of Plos One. Such revisions may necessitate a change in title as well. There are also a series of questions and comments about the site description and methods that need to be addressed to improve quality. And finally, it is crucial that the objectives of the project (TLP evaluation relative to reference and use of CPT to measure success) be clarified throughout the paper and substantiated by the data. Reviewer 3 makes some useful suggestions in this regard. Addressing suggestions from these reviews will greatly improve the clarity of the paper and its potential impact in the field of wetland restoration, while also expanding the scope to be more suitable for the international readership of the journal. | The authors appreciate the Editor-in-Chief’s invitation to revise the manuscript. The comments and recommendations provided have ultimately led to a more clear and concise manuscript. Thank you. |

| **Reviewer #1 Comments (PDF)** | **Authors’ Responses** |
| --- | --- |
| Reviewer #1: Improvement of English is suggested. For example, the article mentions "At the time of investigation" on several occasions. Rewriting of those sentences is suggested. Also, the article has issues associated with same word once UPPER CASE and once LOWER CASE. Some of the references were not written appropriately. Mixing of present and past tense. The pdf should contain the suggested corrections. Please check those. | The authors appreciate the suggested revisions. The major comments have been listed and responded to below while the minor corrects have been adder without a formal response. Thank you. |
| 161. Rewrite this sentence showing the reference somewhere in the middle | The sentence has been rewritten as follows:  “The CPT utilized was specifically developed for use in ultra-soft wetland soils, commonly found in coastal Louisiana, that incorporates both geotechnical parameters (tip resistance, sleeve friction, and pore pressure) and abiotic parameters (soil moisture, electrical resistivity, and temperature) [21].” |
| 182. What does it mean? (referring to sampling frequency) | Sampling frequency is the capacity of the data acquisition system to record measurements. On average, we get roughly 3 measurements each second. |
| 189. Why 4°C, why not the field condition ambient temperature? | The samples were kept at 4°C to preserve the specimen and prevent the roots from decomposing. |
| 455. Conflicting with the Financial Disclosure Statement | The reference to funding within the acknowledgements section has been removed:  “The authors would like to thank support offered by the Engineering Research and Development Center (ERDC) through the USACE Dredged Operations and Environmental Research (DOER) program…” |
| 478. What is the relation of this article with the current study? | The article and citation has been removed from the text. |

| **Reviewer #2 Comments** | **Authors’ Responses** |
| --- | --- |
| Reviewer #2: Overall, I think this is an interesting article that should be accepted for publication in PLOS One pending major revision as detailed in the comments below. The manuscript addresses the impacts on sediment parameters of a thin layer placement. Sea level rise is a challenge faced by wetlands globally, thus evaluating strategies to help marshes keep pace with sea level rise is important. Despite the importance of the topic, this paper needs major revision before being acceptable for publication in PLOS One. The major and minor issues that I found are documented below. | The authors appreciate the comments provided. A detailed response to all comments is below. |
| Introduction: I think that the overall introduction of this paper needs to be broadened in scope, terminology, and literature review to be more applicable to the global audience of PLOS One. a. PLOS One is read by a wide range of scientists, not just engineers so I think that some of the background need broader focus, and I have tried to suggest places below (in specific comments) where I feel those additional references or information is needed. b. A lot of the terminology used (esp. nourishment) is regionally specific. c. Most of the literature discussed is older and focused on just a couple of ecosystems in the United States (Gulf of Mexico and East Coast). This should be broadened to include more recent studies (e.g. Cahoon et al. 2019 Estuaries and Coasts) and more diverse locations (e.g. Thorne et al. 2019 Ecol Eng from CA, Wigand et al. 2017 Estuaries and Coasts). | The authors appreciate the suggestions and have completed major revisions to the introduction section to broaden the scope of studies and remove regional terminologies. In addition, a West Coast, USA study has been added. |
| Methods: Overall, there is a lot of repetitiveness and misplaced information between the Methods and Results. Both sections should be cleaned up and better organized. In addition, the site and experiment description need to clearer as I lost track of what a region versus a site versus a transect versus a TLP area was. a. The figures and tables are emphasized in the sentence structure instead of the result being discussed with a parenthetic reference to the table or figure that presents the data. b. A lot of the text in the Results seems to just describe the numbers from the figures which I found confusing. I could see the numbers on the figures and would prefer to have the pattern described in the Results text. c. Several key methods about vegetation cover and grain size are missing. | The Methods and Results sections have been completed revised to remove redundancy and misplaced information. The transect names and sites have been simplified to reduce confusion. In addition, the Results section has been refocused on patterns and not the specific numbers. Thank you. |
| Figures: I found several of the figures not as clear as could be due to missing labelling or need of additional details. These are discussed in detail below. | The authors have revised problematic figures and captions to be more concise. |
| Throughout the manuscript, I felt as if the parameters (shear strength) measured were done well but then how broadly these could be applied were overstated. CPT strength measurements may correlate with the measured parameters but it is overstated to claim that they predict restoration success or the entire ecological restoration trajectory. I think being clearer in what is measured at the start and what is inferred would improve the manuscript. | The authors appreciate the comment and have performed major revisions to the structure and tone of the manuscript to refocus it to not overstate the application of the cone penetrometer. |
| 59. This is really not establishment of soil strength but redevelopment of (post-TLP) or changes in. | Post sediment nourishment, the strength is establishing and stating it is re-establishing would infer the strength and site are reverting back to a previous state which will not occur. |
| 60. What are establishment markers? This is too vague for an important point in the abstract. | Establishment markers were measurements that can be used to monitor the development of a site like biomass or bulk density. We have changed the term to “performance measures” to try and make it clearer of what these measurements are used for. |
| 60. I think this should read “lower strengths”, not “weaker strengths” which is confusing. | This suggestion has been made to the text. |
| 64-66. (Starting During the construction process): This sentence seems unnecessary and like an anecdote, not an abstract sentence. | “During the construction process” has been removed from the sentence. |
| 61. The root system? Whose roots? Vegetation? | The text has been revised:  “Results show that the nourished area exhibited weaker strengths than the reference sites, suggesting the root system of the vegetation is still establishing.” |
| 64. Trajectories is also too vague. You should state vegetation or belowground biomass specifically because there are a lot of restoration trajectory parameters (e.g. invertebrates, algae) that you are not measuring. | The sentence has been revised as follows:  “The belowground biomass measurements correlated to the CPT strength measurements, demonstrating that shear strength measured from the cone penetrometer could serve as a surrogate to monitor wetland vegetation trajectories.” |
| 74. Habitats should not be plural. | The correction has been made. |
| 76. hurricanes have accelerated | The correction has been made. |
| L. 77. I think is truer if written “loss, leading to need for comprehensive coastal restoration management plans” so this did not always happen. | The authors agree with this comment, however the Introduction has been revised and this statement is no longer in the text. |
| 81-83. This statement is not always true – it is hypothesized that TLP can be conducted without ecological implications but that varies with location and impacts measured and thickness of sediment applied. A more in-depth literature review is required here. This is also a sentence that needs more modern and more geographically varied references (as discussed above). | The authors completely agree that this is not always the outcome but it is the stated definition of TLP and is a main goal of this method. The Introduction has been revised and this statement is no longer in the text. |
| 83-85. There are more than just this method to conduct TLP – if you are going to discuss this you should acknowledge other methods or drop this sentence. | The sentence has been removed. |
| 91-93. This repetitive of earlier paragraph. | The Introduction has been completely revised to reduce the repetitiveness. Thank you. |
| 98. Spartina-dominated | The text has been revised. |
| 99. Where were these experiments? Location and type of marsh would be useful for understanding the context of this reference. | The Introduction has been revised and this statement is no longer in the text. |
| 101-102. You also need to expand this discussion or delete this sentence. | The sentence has been deleted. |
| 103-105. I would split this into two sentences after the reference as right now it has a misplaced modifier. | The Introduction has been revised and this statement is no longer in the text. |
| 106. Delete establishment as I think this is confusing. | Establishment has been removed from the sentence. |
| 108. What do you mean by physical index properties? | The physical index properties of the sediment measurements were bulk density and moisture content. |
| 109. You refer to the dredge outfall, but we need more information. Is this permanent or just for construction? | The dredge outfall location was the site the outlet pipe was stationed to deposit material into the site. After completion of the sediment placement, the pipe was removed. |
| 111-113. We also need more information about the reference site to evaluate its choice as a comparison. It is unfortunate that you are missing sediment cores from there but other site parameters will help justify its choice as a reference site. | The reference site was selected based on areal images over the past 3 decades. The reference site remained unchanged over the decades and assumed healthy and stable. Having cores would have been great but based on our previous experience using the CPT, it is a good indicator of belowground biomass. A sentence has been added explaining this:  “The reference area was assumed as stable (i.e., no discernable deterioration) over the past three decades based on time-lapse aerial images.” |
| 113. This is an example of where I feel the implications of this work are overstated. This will not define wetland restoration success but will instead advance our understanding of how CTP relates to specific biomass parameters. You can make the argument (which you should) that AG and BG biomass are related to important ecosystem development metrics. | The sentence has been revised to state the outcomes more clearly:  “This study is the first to evaluate the benefits of dredged sediment on a wetland using a CPT and traditional trajectory performance measurements to quantify soil strength gain within a wetland nourishment. These findings can serve as a benchmark for future restoration projects by demonstrating useful establishment monitoring practices for coastal stakeholders.” |
| Figure 1. This figure needs larger context. I would like to see the inset be expanded to show more of the East Coast, not just a section of NJ and DE. This is hard for a non-local or global reader to understand. | Figure 1 has been updated. See below: |
|  | |
| 120. On the bay side of what? And from Figure 1, it appears as if is actually on the Oceanside? Please clarify. | The site is located on the bay side of Avalon, NJ. Figure 1 has been revised to show a larger extent to reduce future confusion. In addition, the sentence has been revised:  “The study site is located near Avalon, New Jersey, USA (Fig 1).” |
| 120. The wetland – which wetland? The TLP site or the reference site or the whole thing? | The area contains both the thin layer placement site and reference sites. The text has been revised as follows:  “The sites are within a 17 km^2^ tidal marsh complex adjacent to Great Sound.” |
| 127. When was the area identified as transitioning? The idea of this rapid transition is mentioned several times throughout the paper but not enough detail on the time frame and the observed transition is presented to completely understand. | The area was identified as transitioning over the past 30 years by local practitioners. The text has been revised:  “Wetland degradation of the area was identified in the rapid transition of vegetated marsh platforms to un-vegetated shallow pannes through erosion and vegetation stress over the course of decades.” |
| 131. Onwards in this paragraph: The methods behind these measurements needs to be explained. In fact, these data are repeated in different places. I think the methods for this survey need to be explained in the Methods, and these data need to be presented in the Results. | This section has been revised to address this comment. Thank you for pointing this out to us. |
| 134. This is an example of the terminology confusion – what is areas here? Is this the shallow pannes discussed above (L. 132)? | To limit confusion, the following sentence has been cut form the text:  “Shallow areas were found to be continuously inundated or saturated.” |
| 134. Again my comment about rapid transition. Over what time period? | The area was identified as transitioning over the past 30 years by local practitioners. |
| 144. This seems repetitive of information above or I do not understand the terms for your site versus marsh etc. | The purpose of this sentence is to provide design parameters of the restoration (design fill height) and give an idea of the varying fill thicknesses experienced across the area. |
| 150. Another landscape term is introduced here “cells”. These should appear on the map. | The 2016 nourishment consisted of 5 distinct cells which are areas contained using coir logs and for this study, we only investigated 1 of the 5 cells. To prevent confusion the sentence has been revised as follows:  “Two transects across the nourished wetland were investigated…” |
| 151. I would rename “Interior” | The transects have been renamed to Transects A and B. |
| 153-154: This sentence is confusing. Do both transects begin at the dredge outfall or do they begin at different starting points? Or do you mean that the two transects have different elevations and starting habitats? | Both of the transects start at the dredge outfall and move in different orientations from this point. The sentence has been revised as follows:  “Both transects extended 100 m from the dredge outfall with 20 m sample spacing and were selected to show contrasting starting points on the implications of establishment.” |
| 157. Write out the reference name in this case. | This section has been rewritten based on another reviewer’s comment as follows:  “The CPT utilized was specifically developed for use in ultra-soft wetland soils, commonly found in coastal Louisiana, that incorporates both geotechnical parameters (tip resistance, sleeve friction, and pore pressure) and abiotic parameters (soil moisture, electrical resistivity, and temperature) [21].” |
| 159. I think the more accurate term is abiotic, not ecological. | The correction has been made. |
| 166. What four tests? Are these pilot evaluations? | Four sequential tests were conducted at each sampling site to show repeatability in the tests and provide statistical significance. |
| 174. onwards should be moved to Results. | The authors believe this information is important to understanding the results from the cone penetrometer and should remain within the methodology section since it pertains to data processing. |
| 188. Again what are sites? The TLP versus reference marshes? Or the points along the transect? | These samples are only along the two transects that are within the TLP area. The intro sentence has been revised to reduce confusion:  “Along the transects, soil samples were collected using polyvinyl chloride (PVC) cores and a Russian Peat Corer to validate the CPT measurements with traditional performance measures.” |
| 190. Here is an example of leading with the Figure instead of stating the results and referring to the figure. | Thank you, the text has been revised to follow this format. |
| 195. Was the vegetation rinsed to remove mud? | Yes, the vegetation was thoroughly rinsed to remove the soil. |
| 200-207. This should be moved to Methods | After reviewing all comments, we have decided to remove this section discussing the sediment thickness and subsequent loss from the paper to ensure the main point of this paper is the focus. |
| 209. The amount of elevation loss over what time period? | The elevation loss is shown over a 15-month period and this section has been removed. This section has been removed from the text. |
| 201. Here another landscape term is introduced – region. While these are shown on the map, it is confusing to understand how cell, site, region all fit together in the sampling scheme. | This section has been removed. |
| 219-22. These sentences have a lot of interpretation and belong in the Discussion. | This section has been removed. |
| 223-227. These sentences belong in methods. | This section has been removed. |
| 225. No grain size methods were discussed at all in the Methods. This needs to be added. | The grain size was determined based on ASTM D6913:  “The dredged sediment depth was noted and collected in bags to determine the grain size of the deposited material in accordance with [35].” |
| 227. Post-nourishment | The word “directly” has been removed from the sentence. |
| 227. I am not sure what is meant by an increase? I think it just needs to be reworded to be clearer. | The sentence has been revised as follows:  “The 2016 sampling showed that as distance from the dredge outfall increased, the percentage of fine-grained sediment increased. This is due to coarser material (i.e., sands) falling out of suspension faster than the finer material (i.e., silts and clays).” |
| 231. This belongs in the Discussion, and this is an example of what I find overstated. We don’t know that this is vegetation-driven accretion from your study. | The authors believe this sentence belongs in the Results to serve as a conclusion to the paragraph. The sentence has been revised to not overstate what was not investigated:  “This redistribution of fines could be attributed to vegetation-driven accretion or movement of the sediment due to hydrodynamic processes and bioturbation [13].” |
| Headings of Establishment here confused me: establishment of what? | The heading has been revised as follows:  “*Transect A: Soil Strength*”  And  “*Transect B: Soil Strength”* |
| 234-236. This is already described in the Methods so I think could be deleted. | The sentence has been removed. |
| 235. Vegetation methods need to be described better in Methods (as discussed above) so this should not be “appeared to be” but an actual community analysis or description. This paragraph has lots of examples of leading with the Figure or Table when the results should be summarized. | The majority of vegetation discussion has been removed from the manuscript to focus the discussion on belowground measurements. The belowground biomass process and collection of soil samples has been described in the methodology section. |
| 240. Shear strength increased down the core or over time?  This whole section is difficult for me to read – I think the figures speak for themselves and I would prefer to see the pattern described without the exact numbers. With a well-designed figure, I can read the exact numbers myself. | The authors appreciate the comments and have completely rewritten the paragraph to focus on patterns instead of exact numbers. Thank you. |
| 240. At the time of investigation? Tell us when it was. | These measurements were taken in 2019. To reduce reader confusion, we have revised the sections. |
| Table 1 is not vegetation but AG and BG biomass | The title has been revised as follows:  “Table 1. Site description and belowground characteristics along Transect A. Surface type is Tall-form *S. alterniflora* (Tall), Short-form *S. alterniflora* (Short), and Mudflat.” |
| 268. Again the opening sentences are repetitive of information from earlier and should be deleted. | The sentence has been removed. |
| 296. More attention needs to be paid to the missing data from Reference site. I understand that things happen but it could affect conclusions. | Even though soil cores were not collected from the reference site, the authors believe the CPT measurements are representative since the main comparison being made is in regards to strength. |
| 296. Dominant, not dominate | The correction has been made. |
| Compaction: This section does not contribute much to the manuscript in my mind so either needs to be better linked and connected to the story or deleted. | This section was restructured and rewritten into the discussion section. The authors believe this section provides value because this was a phenomenon noticed while in the field and we were able to clearly pick it up within the CPT measurements. |
| 373. This is missing something…. | The authors believe you are referring to the mention of tip resistance instead of the previously mentioned sleeve resistance. The tip resistance is another measurement collected by the CPT and the compaction was shown clearer using this parameter due to the larger surface area which is why we chose to use it. |
| 381. This is still one of the points on which I am confused. When are we talking about specifically? Why was the site so degraded? Salt pannes can be natural so I am not convinced that the transition to salt pannes alone was bad or a sign of degradation. While I like linking this back to ecological theory, I am not convinced of the stage model as it relates to your data and conclusions. | The discussion section has been restructured and this section is no longer in the manuscript. Thank you. |

| **Reviewer #3 Comments** | **Authors’ Responses** |
| --- | --- |
| This manuscript addresses soil strength and biophysical factors of thin layer placement as a restoration technique for degraded marshes. The manuscript describes the use of a cone penetrometer as a proxy for soil strength along two transects, a heavy machinery impacted zone, and a control area. Overall, the manuscript addresses important data gaps in methodology and marsh restoration trajectories following thin layer placement. However, the manuscript requires additional clarification and text in several locations, and mainly in the discussion section. I am recommending major revisions to this manuscript, which upon completion will provide important new data to the wetland literature. Specific comments are provided below. | The authors appreciate Reviewer #3’s time and comments provided. The provided comments have been taking into account and have ultimately lead to a better manuscript. Thank you. |
| 60. Clarify “traditional wetland establishment markers” to specify belowground biomass, bulk density, and moisture content. Are these parameters only related to wetland establishment, or more broadly applicable to wetland condition? | Establishment markers were measurements that can be used to monitor the development of a site like biomass or bulk density. We have changed the term to “performance measures” to make it clearer of what these measurements are used for. |
| 61. This statement is a little misleading. Based on the site description, large areas were un-vegetated prior to thin layer placement. Post thin layer placement, these areas are beginning to vegetate. Is a comparison between a vegetated control area and an un-vegetated open treatment area valid? | The comparison is between the nourished site which was previously a mix of open water and sparely vegetated sections and section of wetland consisting of both tall- and short-forms of Spartina that have remained stable (unchanged) over the past 3 decades based on aerial images. |
| 80. The use of “nourishment” is also misleading. Are you nourishing elevation? The objective of the thin layer placement is to increase elevation. A secondary effect is nutrient addition (i.e. “nourishment”) from the dredged material. | You are correct, the main purpose of TLP is to increase the wetland surface elevation. The authors believe referring to this TLP as “nourishment” is justified because the majority of literature and government agencies refer to it as so. |
| 85. There are examples of thin layer placement on the West coast, and should be included in this section. Specifically, Seal Beach, CA is an example of a West Coast TLP project. | Thank you for pointing this out; we have added a reference from Seal Beach, CA and revised the sentence:  “Thin layer placement (TLP) is a common restoration management strategy used throughout the Gulf, Atlantic, and Pacific coasts of the United States that focuses on improving biotic and abiotic environmental conditions through pumping hydraulically dredged sediments onto the marsh platform [8-17].” |
| 92. Is there really a significant contribution of organic material from the dredged sediment? | Depending on the area, yes. For example, in an adjacent sediment nourishment in NJ, high concentrations of organic material were encounter (that actually clogged the pipes). |
| 102. Add a transition here; the change in ecological discussion to shear strength is abrupt. | The authors appreciate the comment and have completely restructured and rewritten the instruction section. Thank you for pointing this out. |
| 129. The literature uses “short form” and “tall form” of S. alterniflora. | The correction has been made. Thank you. |
| 154. Establishment of what? | The text has been revised as follows:  “…implications of the soil strength establishment.” |
| 157. Is ultra-soft wetland soils analogous to unconsolidated soils? If so, include unconsolidated soils as an additional term that people are familiar with. If not, define what “ultra-soft” refers to. | The text has been revised to better explain ultra-soft soils:  “These ultra-soft soils have high water content and compressibility, may be underconsolidated, and are undergoing self-weight consolidation [32].” |
| 204. Consolidation of the dredged material is expected. However this is not addressed until much farther in the text. Recommend this information is moved up in the text to clarify that elevation loss was not un-expected. | After reviewing all comments, we have decided to remove this section discussing the sediment thickness and subsequent loss from the paper to ensure the main point of this paper is the focus. |
| 231. Vegetation driven accretion? The accumulation of organic material through vegetation growth redistributes fine materials? More likely redistribution of fine material is through hydrodynamic processes and bioturbation; above ground biomass would increase fine material deposition. Clarification of this statement is needed. | The sentence has been revised as follows:  “This redistribution of fines could be attributed to vegetation-driven accretion or movement of the sediment due to hydrodynamic processes and bioturbation [13].” |
| 265. Table 1. What does Vegetation Depth (cm) describe? The original marsh surface? Clarification is needed. | The vegetation depth is the depth from ground surface that the strength gain of the root system is noted from the cone penetrometer. To reduce future uncertainty, the tables have been revised to read “Root Depth” instead of “Vegetation Depth.” |
| 326. Clarify what “dredge sediment is further established” means. Vegetation establishment within the placement sediment? | Yes that is correct. We believe that as the root system is further developed within the dredged sediment, this will be accompanied by an increase in shear strength. |
| 361. Correct biomass to organic material. | The correction has been made:  “These higher moisture contents are a key indicator of greater biomass establishment in areas receiving less than 10 cm of sediment whereas greater sediment inputs have lower moisture contents due to lower concentrations of organic material.” |
| 365. Tracks of what? | The tracks are from a marsh buggy that was used throughout the construction of the nourishment. |
| 308. The discussion section is very thin. One important point to discuss is open water areas that received dredged material in the context of the vegetated control areas. These are not directly comparable, yet offer an opportunity to discuss the trajectory of an open water area that receives dredge material moving towards shear strength of a vegetated area. Additionally, a discussion of Avalon marsh trajectory is missing, and perhaps is not possible given one sampling date? How does this fit into the conceptual model? Going back to the objectives outlined in 1121-115, was this wetland restoration project a “success”? How do you define success with one sampling date with CPT? There seems to be two competing objectives of this paper: 1. how does the TLP site compare to the reference area and 2. use of the CPT as a methodology to measure restoration success and trajectory. Revise the discussion section, and throughout as needed, to address these two objectives. | The authors appreciate the recommendations. The discussion section has been completely rewritten based on these comments. IN addition, the conceptual model has been removed from the manuscript to focus the paper on the application of the cone penetrometer to belowground characteristics. |
| 387. Does this calculation consider additional potential elevation gains from organic matter accumulation and resulting accretion from healthy vegetation growth? | Yes, this conceptual model includes accretion from both vegetation and sediment, however this section and figure has now been removed from the manuscript. |
